# SMA-TB: study protocol for the phase 2b randomized double-blind, placebo-controlled trial to estimate the potential efficacy and safety of two repurposed drugs, acetylsalicylic acid and ibuprofen, for use as adjunct therapy added to, and compared with, the standard WHO recommended TB regimen

**DOI:** 10.1186/s13063-023-07448-0

**Published:** 2023-06-28

**Authors:** Lilibeth Arias, Kennedy Otwombe, Ziyaad Waja, Nestani Tukvadze, Tamta Korinteli, Tumelo Moloantoa, Kaori L Fonseca, Natasha Pillay, Thabiso Seiphetlo, Dan Ouchi-Vernet, Adrian Siles, Lidia Carabias, Carles Quiñones, Sergo Vashakidze, Neil Martinson, Cristina Vilaplana

**Affiliations:** 1 Unitat de Tuberculosi Experimental, Germans Trias I Pujol Research Institute (IGTP), Badalona, Catalonia Spain; 2 Centro de Investigación Biomédica en Red de Enfermedades Respiratorias (CIBERES), Madrid, Spain; 3 Perinatal HIV Research Unit (PHRU), University of the Witwatersrand, Johannesburg, South Africa; 4 National Center for Tuberculosis and Lung Diseases (NCTLD), Tbilisi, Georgia; 5 Institut d’Investigació en Atenció Primària de Salut Jordi Gol (IDIAPJgol), Barcelona, Spain; 6 Pharmacy department, Germans Trias I Pujol Hospital and Research Institute (HUGTIP-IGTP), Badalona, Catalonia Spain; 7 The University of Georgia, Tbilisi, Georgia; 8 Centre for TB Research, Johns Hopkins University, Baltimore, USA; 9 Microbiology Department, Northern Metropolitan Clinical Laboratory, Hospital Universitari Germans Trias I Pujol, Badalona, Catalonia Spain; 10 Direcció Clínica Territorial de Malalties Infeccioses, Salut Internacional de Gerència Territorial Metropolitana Nord, Badalona, Catalonia Spain

**Keywords:** Tuberculosis, Host-directed therapies, Clinical trial, Infectious diseases, Repurposed drugs, Non-steroidal anti-inflammatories

## Abstract

**Background:**

The duration and regimen of tuberculosis (TB) treatment is currently based predominantly on whether the *M. tuberculosis* (Mtb) strain is drug-sensitive (DS) or multidrug-resistant (MDR) with doses adjusted by patients’ weight only. The systematic stratification of patients for personalized treatment does not exist for TB. As each TB case is different, individualized treatment regimens should be applied to obtain better outcomes. In this scenario, novel therapeutic approaches are urgently needed to (1) improve outcomes and (2) shorten treatment duration, and host-directed therapies (HDT) might be the best solution. Within HDT, repurposed drugs represent a shortcut in drug development and can be implemented at the short term. As hyperinflammation is associated with worse outcomes, HDT with an anti-inflammatory effect might improve outcomes by reducing tissue damage and thus the risk of permanent sequelae.

**Methods:**

SMA-TB is a multicentre randomized, phase IIB, placebo-controlled, three-arm, double-blinded clinical trial (CT) that has been designed in the context of the EC-funded SMA-TB Project (www.smatb.eu) in which we propose to use 2 common non-steroidal anti-inflammatory drugs (NSAID), acetylsalicylic acid (ASA) and ibuprofen (Ibu), as an HDT for use as adjunct therapy added to, and compared with, the standard of care (SoC) World Health Organization (WHO)-recommended TB regimen in TB patients. A total of 354 South African and Georgian adults diagnosed with confirmed pulmonary TB will be randomized into SoC TB treatment + placebo, SoC + acetylsalicylic acid or SoC + ibuprofen.

**Discussion:**

SMA-TB will provide proof of concept of the HDT as a co-adjuvant treatment and identify the suitability of the intervention for different population groups (different epidemiological settings and drug susceptibility) in the reduction of tissue damage and risk of bad outcomes for TB patients. This regimen potentially will be more effective and targeted: organ saving, reducing tissue damage and thereby decreasing the length of treatment and sequelae, increasing cure rates and pathogen clearance and decreasing transmission rates. It will result in better clinical practice, care management and increased well-being of TB patients.

**Trial registration:**

Clinicaltrials.gov NCT04575519. Registered on October 5, 2020.

## Administrative information

We used the SPIRIT checklist when writing our report [1]. The numbers in curly brackets in this protocol refer to the SPIRIT checklist item numbers. The order of the items has been modified to group similar items (see https://www.equator-network.org/reporting-guidelines/spirit-2013-statement-defining-standard-protocol-items-for-clinical-trials/).

**Table:** 

Title {1}	Phase 2b randomized double-blind, placebo-controlled trial to estimate the potential efficacy and safety of two repurposed drugs, acetylsalicylic acid and ibuprofen, for use as adjunct therapy added to, and compared with, the standard WHO recommended TB regimen
Trial registration {2a, 2b}	The study protocol has been registered in clinicaltrials.gov identified with NCT04575519 on 5 October 2020 https://clinicaltrials.gov/ct2/show/NCT04575519
Protocol version {3}	Version 5.0 of 8 September 2021
Funding {4}	This project has received funding from the European Union’s Horizon 2020 research and innovation programme under grant agreement No 847762
Author details {5}	^1^ Unitat de Tuberculosi Experimental, Germans Trias i Pujol Research Institute (IGTP), Badalona, Catalonia, Spain. ^2^ Centro de Investigación Biomédica en Red de Enfermedades Respiratorias (CIBERES), Madrid, Spain. ^3^ Perinatal HIV Research Unit (PHRU), University of the Witwatersrand, Johannesburg, South Africa. ^4^ National Center for Tuberculosis and Lung Diseases (NCTLD), Tbilisi, Georgia. ^5^ Institut d’Investigació en Atenció Primària de Salut Jordi Gol (IDIAPJgol), Barcelona, Spain. ^6^ Pharmacy department, Germans Trias i Pujol Hospital and Research Institute (HUGTIP-IGTP), Badalona, Catalonia, Spain. ^7^ The University of Georgia, Tbilisi, Georgia. ^8^Microbiology Department, Northern Metropolitan Clinical Laboratory, Hospital Universitari Germans Trias i Pujol, Badalona, Catalonia, Spain. ^9^ Direcció Clínica Territorial de Malalties Infeccioses i Salut Internacional de Gerència Territorial Metropolitana Nord. Badalona, Catalonia, Spain.
Name and contact information for the trial sponsor {5b}	Cristina Vilaplana, Fundació Institut d’Investigació en Ciències de la Salut Germans Trias i Pujol (IGTP) Carretera de Can Ruti, Camí de les Escoles s/n, 08916 Badalona, Barcelona, Spain cvilaplana@igtp.cat + 34 93 033 05 27
Role of sponsor {5c}	The sponsor of the CT is the Fundació Institut Germans Trias i Pujol (IGTP), coordinator of the H2020-funded project within this CT is conducted (“A novel Stratified Medicine Algorithm to predict treatment responses to host-directed therapy in TB patients (SMA-TB)” www.smatb.eu, Grant Agreement (GA) 847,762). Contact for the CT sponsor is Dr Cristina Vilaplana, MD, PhD, the project leader of SMA-TB project and acts as coordinating investigator of the CT. She conceived the CT in collaboration with other authors as reported in names and roles of protocol contributors. The funders of the CT had no role in the conceptualization, study design, data collection, management, analysis, and interpretation of the findings. The EC recommended the submission of the protocol for publication and obliges the publication of the results of the CT whenever they become available, *encouraging Open Science* publication.

## Introduction

### Background and rationale {6a}

Tuberculosis (TB) is a chronic, life-threatening infectious disease which caused 1.6 million deaths in approximately 10.6 million people with TB disease in 2021 [2]. Although most patients with TB are concentrated in high TB burden countries in Asia and Sub-Saharan Africa, TB continues to be of concern in high-income settings including Europe [3]. Globally, South Africa is in the top 20 countries with a high TB burden, and Georgia is one of 18 priority countries that bear 85% of the European region’s TB burden [4]. Moreover, the increasing number of patients with multidrug-resistant (MDR) TB poses a problem for national health systems globally, which have to treat the complications associated with MDR-TB, the higher rate of poor treatment outcomes, sequelae, and costs. The duration and regimen of TB treatment are currently based predominantly on the drug sensitivity of the causative *M. tuberculosis* (Mtb) strain [5, 6]. Novel therapeutic approaches are urgently needed to (1) improve outcomes and (2) shorten treatment duration. The TB drug pipeline remains sparse, with repurposed antibiotics (levofloxacin, moxifloxacin, clofazimine, cycloserine, terizidone, imipenem-cilastatin, meropenem) and only 2 new drugs (delamanid and bedaquiline) having been proposed with success in the last 50 years and incorporated in the World Health Organization (WHO) TB treatment guidelines update [5].

There is growing evidence that repurposed drugs can significantly improve patient outcomes, particularly for extensively drug-resistant TB (XDR-TB). Repurposed medicines with proven efficacy in TB or potential efficacy in highly resistant TB cases can fill important gaps in MDR-TB treatment, and are of increasing importance in both trial and operational research for MDR-TB. But they need to be made accessible [7].

Host-directed therapies (HDT) have emerged as a great alternative with the potential to modulate the immune system and improve treatment outcomes while reducing the duration of treatment [8, 9]. Repurposed therapeutics offer a potential shortcut to finding an effective HDT. As hyperinflammation is associated with worse outcomes, HDT with an anti-inflammatory effect might improve outcomes by reducing tissue damage and the risk of permanent sequelae [10]. Anti-inflammatory agents could potentially reduce the duration of TB treatment, increase cure rates and improve long-term outcomes of TB patients, both in DS-TB and MDR-TB. However, the variability between patients should be considered when considering treating patients with HDT, as patients may not all benefit equally. Evaluation of a cheap, globally available HDT to be administered as a co-adjuvant to standard TB treatment to enhance its efficacy is therefore needed [11-14].

Acetylsalicylic acid (ASA) and ibuprofen (Ibu) are non-steroidal anti-inflammatory drug (NSAID), ‘repurposed’ drug with anti-inflammatory effect and potential anti-TB activity that could have benefit if ‘repurposed’ to augment TB treatment. They are both inexpensive over-the-counter medications and are safe to use at recommended doses have been widely used for decades, approved for over-the-counter (OTC) sale without prescription and included in the 18th WHO Essential Medicines List [15].

ASA is a salicylate, which unselectively inhibits the 2 isoforms of COX (COX-1 and COX-2), as other non-selective NSAIDs, but does it irreversibly and has an antiplatelet effect [16]. ASA has been successfully used as acute and long-term treatment of chronic inflammatory diseases with limited adverse effects [17, 18]. Ibu is also a nonselective COX inhibitor that is used for treating pain, fever, and inflammation including painful menstrual periods, migraines, and rheumatoid arthritis [19]. ASA and Ibu were chosen for the potential benefit on TB (based on scientific literature) [20-23], safety profile, price and availability, and major assets when considering an intervention to be globally implemented for the maximum best interest of both the patients and health systems.

### Objectives {7}

The primary objective is to evaluate the potential benefit of ASA and Ibu, used as HDT when administered concurrently with the standard of care (SoC) WHO-recommended TB regimen in DS and MDR TB patients.

### Trial design {8}

This is a randomized, multicentre, phase IIB, placebo-controlled, three-arm, double-blinded clinical trial (CT). A total of 354 evaluable participants will be recruited and followed up (FU), in the ratio of approximately one-third each at the two South African sites (Soweto in Gauteng and Matlosana in Northwest) and one-third at the trial site in Tbilisi, Georgia. Once recruited, patients will be randomized to one of the experimental groups (a) SoC TB treatment + placebo (control group, arm 1), (b) SoC TB treatment + ASA (arm 2), or (c) SoC TB treatment + Ibu (arm 3).

**Fig. 1:**
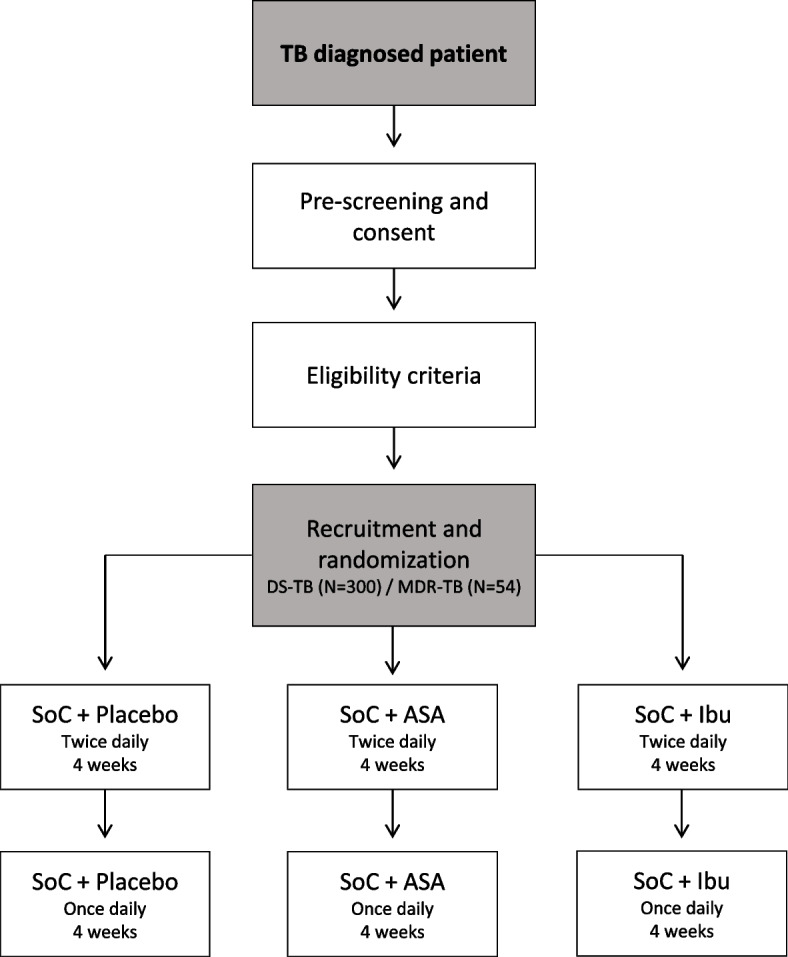
Schematic of the study design

All DS-TB cases (*N* = 300) will receive SoC TB treatment (2HREZ/4HR), consisting an intensive phase of 2 months of daily isoniazid (H), rifampicin (R), ethambutol (E), and pyrazinamide (Z), followed by a continuation phase of 4 months of daily of isoniazid (H) and rifampicin (R). We will also include 54 MDR-TB for exploratory analysis. Only rifampicin monoresistant, isoniazid monoresistant, or rifampicin + isoniazid resistant participants will be included. The study definition of MDR TB will be based on the detection of rifampicin-resistant TB on the Xpert MTB/RIF Ultra (Cepheid, Sunnyvale, CA). The optimal combinatorial treatment regimen according to WHO guidelines and drug availability in the country will be considered as SoC for MDR-TB patients (Fig. 1).

## Methods: participants, interventions, and outcomes

### Study setting {9}

The study is being conducted in the perinatal HIV research unit’s (PHRU) clinical research site at Chris Hani Baragwanath Academic Hospital (CHBAH) in Soweto, the PHRU satellite clinical research site at the Klerksdorp – Tshepong Hospital Complex (KTHC) in the Matlosana Municipality around Klerksdorp, South-Africa, and in the National Center for Tuberculosis and Lung Diseases (NCTLD) of Georgia.

### Eligibility criteria {10}

#### Inclusion criteria

We will recruit adults, patients 18–60 years old with laboratory-confirmed pulmonary TB, regardless of sex and HIV infection status. People living with HIV will be included if their CD4 count is ≥ 100 cells/mm^3^ or they are receiving suppressive ART (viral load at baseline ≤ 400 copies/ml) at the time of recruitment. All will provide their own written informed consent.

#### Exclusion criteria

The presence of major comorbid conditions for which NSAIDs or anticoagulants are therapeutically indicated or could be prescribed while in trial follow-up, or therapeutically contraindicated were the exclusion criteria. We will exclude those who had received TB treatment for > 4 days in the 6 months prior to randomization, women who are pregnant or breastfeeding, people with major alterations in laboratory parameters taken prior to randomization, weight ≤ 45 kg, and/or evidence of excessive alcohol intake. Any other finding which in the opinion of the investigator would compromise the protocol compliance or significantly influence the interpretation of results were also exclusion criteria. All other concomitant medications essential for the participant are permitted except for the listed above and will be properly documented on the CRF. A detailed list of inclusion and exclusion criteria is provided in Table 1.

**Table 1:** Inclusion and exclusion criteria

Inclusion criteria	Exclusion criteria
**1. Adults, 18–60 years of age**	1. Has a **comorbid condition where treatment with ASA, Ibu, or other NSAID is indicated** (e.g. cardiovascular disease, rheumatic fever, chronic pain)
**2. Written informed consent in a language they understand. This includes informed consent to be in the trial and informed consent to collect specimens**	2. People **institutionalized** (incarceration in jail or prison or due to chronic mental illness). If incarcerated during the study, participants may be terminated, and those incarcerated in the first 8 weeks of follow-up will be late exclusions and replaced^a^. Patients either who are planned to be hospitalized or are currently hospitalized while treated for MDR-TB in a TB hospital or ward may be enrolled
**3. Laboratory-confirmed pulmonary TB** (with or without extrapulmonary involvement) defined as a hard copy of a sputum laboratory result that reports *Mtb* detection by a WHO-recommended assay—both rapid molecular assays (MTB/RIF or MTB/RIF ULTRA GeneXpertwith semiquantitative result of ‘medium’ or ‘high’) or mycobacterial culture with subsequent speciation are acceptable as inclusion criteria *Patients who are subsequently found to be culture negative for Mtb at baseline will be late exclusions and not included in the analysis*^a^	**3. Receipt of multi-drug TB treatment** (including rifamycin plus isoniazid preventive treatment regimens) for > 4 days in the 6 months prior to randomization. Participants who have received > 4 days of TB preventive treatment in the month prior to TB treatment initiation will also be excluded
4. Women of childbearing potential (including females < 2 years post-menopausal) **must have a negative pregnancy test at enrolment** *Women who become pregnant during the first 8 weeks of the study will have the study drug stopped immediately. If pregnancy occurs during the first 4 weeks, the participant will be excluded from the analysis*^c^	4. Currently **pregnancy/breastfeeding** *Women who conceive and are found to be pregnant in the first 4 weeks of the trial will be terminated from the trial and excluded from the analysis*^a^
**5. Participant must be willing to have an HIV test done unless there is compelling evidence that the patient is HIV-infected at the time of randomization**. Compelling evidence of HIV infection are as follows: • Hard copy of an unsuppressed viral load • Hard copy of a prescription for antiretroviral therapy • Documentation in the clinical record of two rapid tests positive for HIV infection • Hard copy of a laboratory ELISA or Western blot test positive for HIV	5. **Any of the following laboratory parameters** taken prior to randomization: • Aspartate aminotransferase (AST) or alanine aminotransferase (ALT) > 3 × upper limit of normal (ULN) • Total bilirubin > 2 × ULN • Neutrophil count ≤ 700 neutrophils/mm^3^ • Platelet count < 50,000 cells/mm^3^ • Haemoglobin concentration less than 8 g/dL • Serum creatinine concentration more than twice the upper limit of normal
	6. **Co-treatment** in the 3 months prior to randomization or planned treatment over the course of the trial follow-up **with any one of the following agents**: • Anticoagulant therapy • Immune-modulating therapy (cancer treatments, any oral or daily use of inhaled steroids) • Antacids or proton pump inhibitors—including self-treatment and prescription
	7. History or clinical record of **sensitivity, asthma or allergy** that could be attributed **to NSAIDs**
	8. Weight **< 45 kg** at baseline
	9. A history or **clinical record suggestive of** any of the following in the past 2 years: • Peptic ulcer disease or gastro-intestinal bleeding • Coagulopathy or other bleeding disorder • Renal disease requiring hospitalization—in addition, any prior record at any time of acute kidney injury will be an exclusion criterion • Liver disease requiring further investigation or hospitalization • Underlying cardiovascular disease or risk factors for cardiovascular disease
	10. Patients with **HIV infection if**: • CD4 < 100 cells/mm^3^ (taken within 3 months prior to consent) • If on ART, viral load > 400 copies/ml) (taken within 3 months prior to consent) • If not on ART, either in the opinion of the attending doctor or according to local ART guidelines, the patient should initiate ART during the 8-week initial placebo or NSAID treatment phase
	11. **Alcohol abuse**: potential participant either self-reports or in the investigator’s opinion that the patient drinks more than an average of four units/day over a usual week or is a binge drinker (men: 5 or more drinks; women: consume 4 or more drinks, in about 2 h)^b^
	12. Major co-morbid conditions or **any other finding which in the opinion of the investigator would compromise the protocol compliance or significantly influence the interpretation of results**

^a^Late exclusion criteria: These are criteria which become apparent after randomization (and after likely receipt of the study drug). Where possible, participants who are late exclusions during the initial 8 weeks placebo or NSAID treatment phase will be replaced by another eligible participant

^b^1 unit of alcohol is equivalent to 8 g of ethanol, the amount that can be processed by the average adult in an hour (UK specifications). One tot (25 ml) of alcoholic spirits, half a glass of wine, and 284 ml of beer are roughly equivalent to one unit of alcohol

^c^In South Africa, pregnancy tests will be done at every visit for the first 8 weeks due to high rates of pregnancy

### Who will take informed consent? {26a}

Recruiters will engage with potential participants who have laboratory confirmation of TB with positive sputum GeneXpert and who either have yet to initiate TB treatment or who have taken less than three doses of TB treatment at the time of screening, for this episode of TB disease. Patients diagnosed with TB in clinics will be approached by a recruiter to assess their interest in volunteering for a clinical trial. Eligible individuals will be invited to attend the research clinic to obtain informed consent and further assessment of eligibility. Written informed consent to participate in the study, using institutional review board (IRB)-approved consent forms, will be obtained by trained study personnel prior to performing any study-specific procedures.

### Additional consent provisions for collection and use of participant data and biological specimens {26b}

Additional informed consent will be provided to the participants for storage and use of leftover samples and data for future research, use of blood samples for genetic testing, and HIV testing.

### Interventions

#### Explanation for the choice of comparators {6b}

All patients will be treated with the standard of care (SoC) WHO-recommended TB. The difference between arms will be the addition or not of co-adjuvant treatment with interventional drugs. The placebo group will be used as a control group.

#### Intervention description {11a}

Newly diagnosed, pulmonary TB patients enrolled will be randomized in a 1:1:1 ratio, according to an allocation sequence generated prior to first recruitment (Fig. 1) and not shared with investigators or clinical sites. Allocation to study arms will use a block randomization strategy, stratified by site into one of the following 3 arms, to receive:

1. SoC TB treatment + placebo twice daily during the first 4 weeks of TB treatment followed by placebo once daily for an additional 4 weeks (control group) (arm 1)
2. SoC TB treatment + acetylsalicylic acid administered orally in a gastro-resistant tablet with a dose of 300 mg twice daily during the first 4 weeks of TB treatment followed by a reduced dose of acetylsalicylic acid 300 mg once daily for an additional 4 weeks (arm 2)
3. SoC TB treatment + ibuprofen administered orally in a film-coated tablet with a dose of 400 mg twice daily during the first 4 weeks of TB treatment followed by a reduced dose of ibuprofen 400 mg once daily for an additional 4 weeks (arm 3)

#### Criteria for discontinuing or modifying allocated interventions {11b}

##### Treatment interruptions

Missed doses of IP up to a maximum of 3 doses will not be replaced at the end of the first month when twice daily doses of therapy are administered. However, if > 3 but < 14 doses are missed, the missing doses will be replaced before the participant is moved to month 2 (daily dosing). The schedule of evaluations (and planned safety assessments) remains unchanged. A similar process will be completed for the second month of IP: if a participant does not receive > 3 consecutive doses but < 14 doses, the treatment will be continued until at least a total of 28 doses of IP at the reduced dose have been taken. However, if > 14 days of treatment are missed the treatment will be halted at the originally planned time. Missed TB drug doses will be replaced according to local guidelines.

##### Discontinuation and withdrawal criteria

Participants are free to withdraw from participation in the study at any time upon request or an investigator may terminate their participation. Any discontinuation or withdrawal will be properly documented. Discontinued patients will not be replaced unless they are discontinued during the initial IP treatment phase. The investigator and the sponsor have the right to discontinue the study at any time for reasonable medical and/or administrative reasons and reasons documented appropriately.

#### Strategies to improve adherence to intervention protocols and any procedure for monitoring adherence {11c}

Doses of study drug will be given as directly observed therapy (DOT) by study personnel or by a health care worker or lay treatment supervisor who is aware of the study protocol and trained regarding the study protocol according to the standard practices of each study site. Alternatively, doses of study drugs can be given via DOT by a family member or employer who has been trained by the study team. For this study, DOT dosing implies that the dose has been taken, swallowed and retained for at least half an hour after ingestion. Study operating procedures will detail documentation of DOT and also processes to follow for vomiting of doses.

Adherence will be confirmed by review of the participant’s treatment card, and retraining of the DOT provider will be provided if the treatment card is poorly completed. Adherence will be defined as the number of prescribed doses taken. DOT may be administered at the TB clinic or other health care facility, or, with the participant’s permission, at the participant’s residence, workplace, or other mutually agreed upon location convenient for the participant. Video DOT—where participants video themselves with their phone taking their tablets and then WhatsApp the video to the research site—will also be acceptable, provided the participant concurs and understands the process.

DOT of the first daily dose should occur at least 5 days a week during the first 8 weeks of treatment with weekend doses given as self-administered treatment (SAT). No more than 3 consecutive doses of self-administered treatment (SAT) will be allowed. All doses taken will be recorded on a treatment card and reviewed by the study staff at the next scheduled visit.

#### Relevant concomitant care and interventions that are permitted or prohibited during the trial {11d}

Patients with co-treatment in the 3 months prior to randomization or planned treatment over the course of the trial follow-up with anticoagulant therapy and immune modulating therapy (cancer treatments, any oral or daily use of inhaled steroids and/or antacids, or proton pump inhibitors—including self-treatment and prescription) will be excluded. As well as those who have a comorbid condition where treatment with ASA, Ibu, or other NSAID is indicated (e.g. cardiovascular disease, rheumatic fever, chronic pain).

All other concomitant prescription and over-the-counter medications taken during study participation will be recorded on the case report forms (CRFs) at baseline and at each follow-up visit. Medications to be reported in the CRF are concomitant prescription medications, over-the-counter medications, and non-prescription medications.

#### Provisions for post-trial care {30}

All participants who withdrawn consent or are terminated by the principal investigator will be monitored for safety even after they withdraw from the study. Trial insurance coverage in compliance with the local regulations will be obtained.

### Outcomes {12}

The following are the primary endpoints:

- Time to ≥ 67% sustained reduction in TB score over the course of TB treatment
- Hazard ratio for stable culture conversion (SCC) (≥ 2 consecutive negative cultures for Mtb on specimens taken ≥ 4 weeks apart during the first 24 weeks of TB treatment)

The following are the secondary endpoints:

- Hazard ratio for SCC at weeks 8 and 16 after treatment start
- Proportion of patients with improvement or resolution of clinical signs and symptoms at EOT (TB score)
- Proportion of patients with improvement of lung function impairment as change from baseline at weeks 8 and 24 and EOT in the 1-s forced expiratory volume (FEV1) expressed as FEV1
- Improvement in chest X-ray (CXR) (measured with the BCN-SA score) using the X-ray taken at baseline as the comparator compared with subsequent X-rays over the course of TB therapy: at week 8 and week 24 and for MDR-TB patients at EOT
- Number of patients with improvement of health-related quality of life (HQoL) comparing baseline measure with that over the course of therapy at week 8 and week 24 and for MDR-TB patients at EOT
- Improvement in modified TB score from baseline to end of treatment

The following are the safety and tolerability endpoints:

- Safety: the proportion of participants with serious adverse events (SAEs) between each intervention arm and the control group.
- Tolerability: the proportion of patients in each arm who permanently discontinued either ASA/Ibu/placebo and/or had a TB treatment interruption for longer than seven days/doses, prescribed either by a listed investigator or a non-study physician up to 2 weeks after scheduled or unscheduled permanent discontinuation of ASA/Ibu/placebo. This will be reported as a composite measure of both but each constituent of this endpoint will be reported separately.

The following are the exploratory endpoints:

- A variety of human biological specimens will be collected to identify novel host and pathogen biomarkers
- To develop and test novel composite markers that include clinical, quality of life, digital, and other biological markers of TB treatment response using machine learning techniques

### Participant timeline {13}

The schedule of events with planned visits and study procedures according to the SPIRIT format can be found in Table 2.

**Table 2:** Schedule of events with planned visits

Time point (weeks)	Screening and BL	Intervention (SoC + placebo/ASA/Ibu)								DS and MDR follow-up ON Rx						MDR/Rx treatment extension	Final visit end of FU^c^
	*** − 7 to 0 days***	***1***	***2***	***3***	***4***	***5***	***6***	***7***	***8***	***9***	***10***	***12***	***16***	***20***	***24***^a^	***Extra visit at the completion of TB treatment***^b^	***6 months after the treatment completion date***
**TB score**	X	X	X	X	X	X	X	X	X	X	X	X	X	X	X		
**Eligibility screen**	X																
**Informed consent**	X																
**Randomization**	X																
**Clinical examination**	X	X	X		X				X			X	X	X	X	X	X
**HIV test if required**	X																
**CD4 and HIV RNA**	X																
**Pregnancy test if female**	X				X				X								
**Sputum culture**	X	X	X		X		X		X			X	X	X	X	X	X
**12-lead ECG if MDR**	X				X				X								
**Pulse oximetry, Hb, symptom check, weight, abdominal circumference, MUAC, and anonymized photo**	X	X	X	X	X	X	X	X	X	X	X	X	X	X	X	X	X
**Chest X-ray**	X								X						X		
**Spirometry**	X								X						X		
**HQoL questionnaires**	X				X				X						X		X
**Sample collections for host and pathogen biomarkers**	X	X	X		X				X						X	X	X
**Safety evaluation**	X	X	X		X		X		X			X	X	X	X	X	X
**Safety lab test**	X	X	X		X		X		X			X	X	X	X	X	X
**PK studies**^d^			X									X					
**Adherence check**		X	X	X	X	X	X	X	X			X	X	X	X	X	X

^a^In DS-TB patients, week 24 coincides with treatment completion

^b^An extra visit will be performed at the end of treatment for the MDR-TB group. If TB treatment is extended for more than 15 days in the DS-TB group, an additional treatment completion visit will be conducted

^c^All patients will be followed-up for 6 months (24 weeks) after treatment completion, to investigate relapse

^d^PK studies will be performed on selected participants in South Africa only

### Sample size {14}

Ordinarily, TB treatment lasts for 24 weeks. In our study, the intervention medication will be provided for 8 weeks; it is relatively cheap, of low risk and available over the counter. Thus, everyone is likely to improve. In our experience, most of the improvement occurs by the first 3 months of treatment. Thus, the assumption of a 67% reduction in the TB score is plausible [24, 25]. As a result, we chose a conservative effect size estimate of at least 20% because it may be relevant due to the anticipated benefit of treatment and fewer anticipated adverse events.

The trial sample size was powered for DS-TB patients only for the primary objective only, assuming a TB score reduction of at least 67% by week 15 of FU although our primary outcome allows this to be achieved to the end of TB treatment. The sample size was determined using the survival analysis approach assuming that at least 50% of the participants in the control arm and 70% in the intervention will attain the desired reduction in TB score. Using the anticipated TB reduction rates of 50% and 70%, we estimated the exponential hazards of TB reduction in both the intervention and control arms. Assuming 90% power to detect a minimum difference of 20% in the TB reduction score in favour of the intervention arms, a 15% anticipated loss to follow-up, 2 years of accrual, and 1 year of follow-up, at least 91 participants will be enrolled per arm (total across all arms = 273). Because there is little data on which to estimate relative reductions in TB score, we have further escalated the sample size to 300 (approximately 100 per arm) DS-TB patients. The sample size was estimated using the PROC POWER function of SAS Enterprise Guide 7.15 (SAS Institute Inc., Cary, NC, USA). We will also include 54 MDR-TB patients. These patients have not been included in the sample size estimations and will not be included in the formal assessment of primary outcomes, but will be compared by their clinical responses to treatment to their DS peers, and their biomarker responses to DS-TB patients by arm.

Accrual rates will closely be monitored by the statistics and data management centre (SDMC) to ensure that each site is recruiting as per the study plan. In the event that accrual rates are lower than expected in some sites, the SDMC will recalculate the sample size to ensure appropriate redistribution that introduces no bias.

### Recruitment {15}

Patients with newly diagnosed TB will be identified by recruiters at each site—both in-patients and out-patients newly diagnosed with TB will be screened. We anticipate that most eligible patients will have a hard copy of a positive, sputum Xpert result, but there may be some identified by having a sputum culture positive for Mtb. We anticipate that each study site will recruit at least 50 DS-TB patients. We will attempt to ensure similar numbers of DS-TB participants are recruited at each site but in the event a study site was unable to complete the full recruitment of 100 DS-TB participants per site other sites would competitively enrol until the sample size is met.

### Assignment of interventions: allocation

#### Sequence generation {16a}

Randomization was stratified by site and by whether the patient had DS-TB or MDR-TB in blocks of 9 for the DS-TB and in blocks of 6 for the MDR-TB patients. Each block had the treatments assigned randomly with the guarantee that the three treatment arms are evenly distributed. If eligible and informed consent is obtained, patients will be randomized 1:1:1 into one of the 3 arms. Patient ID was preassigned to a kit before randomization and the last two numbers of the patient identifier matched with the kit’s number to avoid possible errors.

#### Concealment mechanism {16b}

The random allocation sequence remains concealed from the clinical sites and those enrolling patients into the study.

#### Implementation {16c}

Recruiters will engage with potential participants who have laboratory confirmation of TB and who either have yet to initiate TB treatment or who have taken less than three doses of TB treatment at the time of screening, for this episode of TB disease. After the eligibility criteria have been confirmed, the central data centre will receive age, gender, site, and the screening identity number (SID) and will provide a pre-specified randomized participant identification number (PID) that corresponds to a repackaged and relabelled study drug kit in the site pharmacy.

### Assignment of interventions: blinding

#### Who will be blinded {17a}

ASA, Ibu and placebo were obtained by IGTP for all sites and were repackaged and relabelled with a study ID according to the randomization schedule, and sent to sites. Repackaged Ibu, ASA, and placebo are identical in shape, size, and colour.

Both the investigator and participant will be blinded to the treatment regimen. Two independent people will be unblinded for the study and will be the designated people to repackage the investigational products and kept unblinded randomization list.

#### Procedure for unblinding if needed {17b}

Neither clinicians nor patients are unblinded to treatment received unless there is a specific indication to unblind. For any major reason for unblinding a participant allocation while on the trial (i.e. SAE), the clinical context and reason will be reported, the unblinding will be allowed following a randomization and blinding SOP, and the pharmacist will provide the treatment allocation for that participant only.

### Data collection and management

#### Plans for assessment and collection of outcomes {18a}

At the first visit after obtaining informed consent demographic, medical history including concomitant medications are collected as well as vital sign assessment and full physical exam conducted. For women, a pregnancy test is performed, and they are asked for the type of birth control used. At the same visit, lab safety tests, chest X-ray, and HIV testing are also performed; sputum specimens are obtained for GeneXpert MTB/RIF or MTB/RIF ULTRA; and Ziehl–Neelsen staining and cultures on solid and liquid media. A 12-lead ECG test is performed on MDR TB participants.

If inclusion and exclusion are met, at baseline and at the different time points (Table 2), weight and height are recorded for BMI calculation and spirometry is performed. Finally, HQoL questionnaires are administered and safety evaluation is conducted, with adverse events (AE) reviewed at each study visit.

Data from the study is recorded in the created ad-hoc CRF. The recruiting sites use paper source documents to record the data and periodically enter it into the eCRF created in the REDCap system of the WHC.

#### Plans to promote participant retention and complete follow-up {18b}

DOT may be administered at the TB clinic, other health care facilities, or other locations mutually agreed convenient for the participant. Video DOT and SAT during the weekend will also be acceptable. TB treatment may be self-administered or given as DOT after the participant has completed the intervention phase of the study (after 8 weeks).

All FU visits while on TB treatment will be within a visit window of ± 2 days; after TB treatment, visit windows will be ± 12 days. Participants will not be paid for their participation but will receive compensation for their transport and time as per each country’s regulatory guidelines.

#### Data management {19}

A paper CRF will be created ad hoc and filled in for each patient included with all the data required: project’s code (SMA-TB-XXX), clinical characteristics, and monitoring data. In order to comply with the patients’ rights of confidentiality, this form will be pseudonymized, without the name and surnames of the patient and where only the project’s code will be recorded, together with all the clinical data associated to the specific case. In order to comply with the ethics requirements of samples’ collections, both documents will be kept as 2 different documents and stored in 2 different computers.

All the data collected and intended to process will be relevant and limited to the purposes of the SMA-TB project (in accordance with the ‘data minimization’ principle). Source data verification (a comparison of the data in the CRF with the subject’s laboratory test results and other source documents) will also be performed.

Authorized representatives of the regulatory authority may visit the centre to perform inspections, including source data verification. Clean File for the final database will be declared when all data have been entered and a quality check on a sample of the data has been performed. The database will be locked after Clean File has been declared and data extracted for statistical analysis. Study committee meetings will be held as needed prior to or during the study. The medical, nursing, and other staff involved in the study will receive proper education/information on how to conduct the study according to the protocol.

#### Plans for collections, laboratory evaluation, and storage of biologicals specimens for genetic or molecular analysis in this trial/future use {33}

Specimens of a variety of human body fluids (blood, urine, and EBC) and mycobacterial isolates are being collected at baseline; weeks 1, 2, 4, 8, and 24 (end of treatment), and 6 months after the end of treatment. These samples will be used to identify novel biomarkers of TB treatment and HDT responses (exploratory endpoints).

#### Confidentiality {27}

All data will be coded to preserve patients’ identity, according to the Regulation (EU) 2016/679 of the European Parliament and of the Council of 27 April 2016 and equivalent local regulations in South Africa and Georgia on the protection of natural persons with regard to the processing of personal data and on the free movement of such data (as South Africa’s Protection of Personal Information Act 2013 (POPIA)). The clinical history information of patients enrolled will be strictly confidential and their identity will be kept anonymous. For each patient, a pseudonymized form created ad-hoc for this project with a sequential code for each patient will be generated as SMA-TB-XXX that be used to match the patient’s samples with clinical data without the need to use the patient’s name and surname. This form will be to be kept together with the medical record of each patient as the hospital records copy. All data collected and results from the study will be introduced in the REDCap system and will be used for scientific research purposes only, always handled by specialized personnel. Only persons involved in the study and after prior training will receive access to the data.

## Statistical methods

### Statistical methods for primary and secondary outcomes {20a}

For socio-demographic comparisons, descriptive statistics such as median and interquartile ranges will be determined for continuous variables whereas frequencies will be determined for categorical measures. Comparisons between continuous measures will be conducted using the Kruskal–Wallis non-parametric.

For the primary objectives, we will determine the time to a 67% reduction in TB score for each person in each arm. The hypothesis of no difference to the time to a 67% reduction in TB score between each treatment arm and the control will be using the Kaplan–Meier test restricted to time while receiving SoC TB treatment. The hypothesis of no difference in the hazard for stable culture conversion between the treatment and control groups, in the first 24 weeks, will be evaluated using the Cox proportional hazards regression model.

For the secondary objectives, the Kaplan–Meier test will be used to compare the time to stable culture conversion at weeks 8 and 16. The proportion of patients with improvement or resolution of clinical signs and symptoms at the end of treatment will be compared between the groups using the chi-square or Fisher’s exact tests as appropriate. Differences in FEV1 between study arms will be compared using linear mixed modelling. The differences in BCN-SA and health-related Quality of Life Questionnaire scores over time will similarly be compared. All statistical analysis will be conducted in the SAS Enterprise Guide 7.15 and STATA 15 software.

### Interim analyses {21b}

The DSMB will review the proposed protocol prior to its finalization—including scheduling interim analyses and stopping rules. Once a sufficient number of patients have a final primary outcome interim analyses of the outcomes will be shared with the DSMB only, retaining blinding of the investigator team.

### Methods for additional analyses {20b}

Predictive analytic approaches using regularization approaches will be used to evaluate the factors associated with novel composite biomarkers.

### Methods in analysis to handle protocol non-adherence and any statistical methods to handle missing data {20c}

Incomplete data will be evaluated using multiple imputation techniques assuming a missing at random.

### Plans to give access to the full protocol, participant-level data, and statistical code {31c}

The data collected in a database will be kept for a period of 25 years, as required by the EU Clinical Trial Regulation No 536/2014 and complying with all applicable local South African and Georgian regulatory requirements. A data management plan (DMP) was issued for the SMA-TB project, coordinated by the Data Protection Officer of the IGTP, including which data generated by the action, whether and how it will be made accessible and how it will be maintained and preserved.

We plan to include open access to at least some of the anonymized data generated within this trial, for use by others in a secure data repository bug will be guided by local to ethics requirements and in accordance with the FAIR principles. Access to the data will need approval by the investigator and collaborators, for a project that has been granted with ethical approval. If considered appropriate, data and samples could be used (secondary use) for studies related to biomarkers of TB response, promoted by the sponsor or SMA-TB Consortium partners (https://cordis.europa.eu/project/id/847762/).

### Oversight and monitoring

#### Composition of the coordinating centre and trial steering committee {5d}

The trial’s sponsor and the Coordinating Centre will be the IGTP, accredited as a health research centre of excellence by the Instituto Carlos III, and it is part of the network of clinical research units and clinical trials SCReN (Spanish Clinical Research Network https://www.scren.es) that provides support for science and technology in health to encourage independent research in the Spanish and European networks. Each one of the countries conducting the CT, South Africa and Georgia, has its own national principal investigators (PI), working closely to ensure the success of the trial, and each one of the sites has its sites PI. The day-to-day work is supported by recruiters to assess their interest in volunteering for clinical trial and trained study personnel which will obtain written informed consent, using IRB-approved consent forms, perform any study-specific procedures, contact and obtention of approvals from regulatory health authorities and/or ethics committee.

The sponsor designee (CT coordinator) will meet with national PIs and sites PIs routinely every 15 days to provide support for the trial, and more often if needed. Furthermore, the CT is monitored by two independent clinical research associates, one in each country involved, that will report to the sponsor.

A trial steering committee (TSC) comprised 6 senior researchers of internationally repute in the field: the chair, the coordinator, the national PI trialists, and 2 independent researchers not involved in the trial with no competing interests was set up. All major strategic decisions of the CT go through the TSC. The committee meets once a year and more often as needed. Charter for TSC has been issued and filed under the Trial Master File. The CT has the involvement of patients through the local Community Advisory Board (CAB) which are different TB stakeholders and representatives of the TB community. CAB in South African sites assists and advises researchers, informs, and assists participants and the wider community and helps to educate communities on HIV, TB, and clinical research. In Georgia, the NCTLD has been implementing the Community Engagement (CE) to ensure the effective involvement of the community in TB clinical research in order to build and strengthen relationships between researchers and local communities including TB affected population.

#### Composition of the data monitoring committee, its role, and reporting structure {21a}

An independent data safety management board (DSMB) was constituted before the start of the study to provide oversight. It is comprised of 4 members including two TB experts, one clinical research specialist, and one trial statistician. The DSMB meets annually and whenever required and reviews preliminary safety and efficacy data. The DSMB advises on the enrolment’s discontinuation and treatment for arms in which safety or efficacy appears likely to be worse than in the control arm and makes recommendations to the TSC. Charter for DSMB has been issued and filed under the Trial Master File.

#### Adverse event reporting and harms {22}

The AEs recorded during the study period will be graded according to the Division of AIDS Table for Grading the Severity of Adult and Pediatric Adverse Events. All abnormalities occurring during the study period will be recorded in the source documents and eCRF stating the causality of the finding, and patients will be invited for a complete evaluation. The following adverse events must be reported on an adverse event report form: any grade 3 or higher adverse event, new medical diagnosis regardless of grading/worsening of pre-existing diagnosis, study drug discontinuation due to an adverse event, pregnancy, any event that is considered to be probably related to study drugs regardless of grading, and lab values that are considered clinically significant by the investigator. Only AEs reported on an AE form will be included in the analysis. Temporary and permanent discontinuation of treatments will be fully documented in the source document with reasons.

Unanticipated problems (UP) involving risks to participants or others to include, in general, any incident, experience, or outcome that meets all of the following criteria: unexpected in terms of nature, severity, or frequency; related or possibly related to participation in the research; and suggests that the research places participants or others at a greater risk of harm (including physical, psychological, economic, or social harm) than was previously known or recognized.

All AE and UP will be reported. SAEs will be immediately reported to the CT site PI and coordinator. The latter will notify the event to the DSMB, providing a description of the AE in sufficient detail to allow for a complete medical assessment of the case and independent determination of possible causality who will take the correspondent decisions. In addition to the expedited reporting of SAEs, the sponsor will submit, once a year throughout the CT, a safety report to the Institutional Ethics Committee and DSMB. In addition to the expedited reporting of SAEs, the sponsor will submit, once a year throughout the CT, a safety report to the Institutional Ethics Committees and DSMB.

#### Frequency and plans for auditing trial conduct {23}

Site monitoring will be conducted to ensure that human subject protection, study procedures, laboratory procedures, study intervention administration, and data collection processes are of high quality and meet the sponsor, the sites, and regulatory guidelines and that the study is conducted in accordance with the protocol and site SOPs. Site monitoring will be performed according to details in a written clinical monitoring plan by a South African- and Georgian-based Independent Clinical Research Associate (CRA), based on the developed monitoring strategy taking into consideration the enrolment status, data quality, and protocol compliance. Monitoring tasks will be performed in accordance with the protocol-specific requirements, site SOPs, the Integrated Addendum to ICH E6(R1), Guideline for Good Clinical Practice ICH E6(R2), and applicable regulatory requirements.

#### Plans for communicating important protocol amendments to relevant parties {25}

If the protocol must be altered after its signature, the modification will be discussed and approved by the principal investigators and the sponsor. All substantial amendments will be submitted to the Ethics Committee and to the competent authorities for approval. Non-substantial amendments will be submitted to the authorities and ethics committees as notification, i.e. approval is not needed.

#### Dissemination plans {31a}

The results of the trial will be submitted for publication to an open-access peer-reviewed scientific journal and posted in the ClinicalTrials.gov database. Preliminary and final research data will be openly accessible in accordance with the FAIR principles, and results shared with the scientific community through conferences and other relevant events, and to the stakeholders.

## Discussion

SMA-TB is a multicentre, phase 2b, randomized, double-blind, placebo-controlled clinical trial to estimate the potential efficacy and safety of two repurposed drugs, ASA and Ibu, for use as adjunct therapy added to, and compared with, the SoC WHO-recommended TB regimen.

Innovative efforts to identify novel TB treatment regimens include investigational new products not yet licenced, while repurposed drugs offer an opportunity [8]. Despite the great advances, mainly through access to chemotherapy, treatment options for many patients remain very limited, especially for patients suffering from MDR-TB forms. A low-cost intervention having an impact on decreasing treatment length, morbidity, and mortality without increasing drug resistance would be an enormous help to TB patients [26]. Several drugs have been considered HDT in TB, most of them based on in vitro testing and/or theoretical concepts, few being approved by the regulatory bodies, or some having severe AE. In this context, different HDT with different potential acceptability and safety have been proposed to treat TB. Among them, repurposed drugs offer an opportunity to shortcut the licensing pathway [8]. The preclinical studies published in the literature suggest that modulating the immune response involving inflammation signalling might have a positive or negative effect depending on the patients’ disease stage and status [12].

In the context of South Africa, Soweto and Matlosana are 160 km apart and annual TB incidence in the Dr. Kenneth Kaunda District, within which Matlosana is located, is 800/100,000; approximately twice that of the Johannesburg Metropolitan Council within which Soweto is located. Both sites have similar HIV prevalence (~ 30% in pregnant women). PHRU has extensive experience in conducting clinical trials of tuberculosis treatment and has support functions for regulatory, data management, and a research pharmacy currently overseeing ~ 30 clinical trials.

In Georgia, HIV infection rates are low, but there is a high percentage of strains resistant to more than two common TB drugs (MDR/XDR-TB). NCTLD is a lead facility for TB control, which administers and implements the National Tuberculosis Program, aimed to decrease the spreading of TB in Georgia. The NCTLD is subordinate to the Ministry of Labor, Health and Social Affairs, which in general is responsible for TB control in the country. TB management actions are fulfilled through a network of specialized TB service institutions and primary health care services. TB programme has a vertical structure and consists of three levels: (I) national level—NCTLD; (II) regional level—regional TB facilities including TB dispensaries in Tbilisi; and (III) district level—TB units and PHC facilities. In the past 2–3 years, the building of the NCTLD has been renovated, including MDR-TB and departments for sensitive TB treatment and the overall environmental improvements are sorted.

SMA-TB will provide proof of concept (POC) of the HDT as co-adjuvant treatment, and it will identify the suitability of the intervention for different population groups (different epidemiological settings and drug susceptibility). By using two cheap NSAIDS (ASA and Ibu), worldwide available and considered an essential medicine by the WHO and for which we have already done the POC in preclinical studies [12], in case of proven useful, it will be easy to implement this by national health systems and at low cost.

In summary, this regimen will potentially be more effective and targeted, organ saving, reducing tissue damage, and thereby decreasing the length of the treatment and sequelae, increasing the cure rates and pathogen clearance, and decreasing the transmission rate. It will result in better clinical practice, care management, and increased well-being of TB patients. It will also have an impact on the quality of health provided by Health Care Systems: contributing to diminishing the disease burden in individual patients and decreasing the cost per treated person (in €, can achieve an impact on direct costs: hospitalization and outpatient visits; and on indirect costs: productivity loss because of TB or its sequelae).

Even in the case of not finding the intervention superior to the standard treatment alone, the SMA-TB CT will collect a large number of samples and data, which will be further reused by the SMA-TB Consortium partners for investigating and clinically validate biomarkers and link them to clinical, HQoL and social data thanks to Data Science Strategies and Network-based mathematical modelling. The resulting stratification algorithm generated is expected to identify patients at risk of negative outcomes, model treatment response, to predict treatment efficacy/toxicity and to propose personalized options during clinical TB management.

### Trial status

The study protocol was registered in ClinicalTrials.gov identified with NCT04575519 on 5 October 2020. The current trial protocol version is 5.0, issue date: 6 August 2020. Recruitment started on March 2021. The CT, originally scheduled to conclude by June 2024, has been influenced significantly by the COVID pandemic, as highlighted in scientific literature and acknowledged by the WHO [2, 27]. The impact of the pandemic on TB diagnosis has consequently affected the recruitment rate and posed challenges to the trial’s timeline. Budget constraints are also placing additional pressure on the recruitment period and the overall progress of the SMA-TB project. By 6 June 2023, 216 patients have been enrolled, reaching the 61%.

## Data Availability

Any data required to support the protocol may be supplied on request for a specific project with an Ethics Committee approval.

## Abbreviations

AE: Adverse event
ASA: Acetylsalicylic acid
ART: Antiretroviral therapy
CAB: Community advisory boards
CRF: Case report forms
CHBAH: Chris Hani Baragwanath Academic Hospital
CRA: Clinical research associate
CT: Clinical trial
DSMB: Data safety management board
DOT: Directly observed therapy
DS: Drug-sensitive
EOT: End of TB treatment
eCRF: Electronic case report forms
E: Ethambutol
FEV1: Forced expiratory volume
FU: Followed-up
HDT: Host-directed therapies
HQoL: Health-related quality of life
H: Isoniazid
Ibu: Ibuprofen
IP: Investigational product
KTHC: Klerksdorp-Tshepong Hospital Complex
Mtb: *M. tuberculosis*
MDR: Multidrug-resistant
NSAID: Non-steroidal anti-inflammatory drugs
NCTLD: National Center for Tuberculosis and Lung Diseases
OTC: Over-the-counter sale
PI: Principal investigator
PID: Participant identification number
PHRU: Perinatal and HIV research unit
POC: Proof of concept
R: Rifampicin
SIC: Screening identity number
SoC: Standard of care
SAT: Self-administered treatment
SCC: Stable culture conversion
SAE: Serious adverse event
SMDC: Statistics and Data Management Centre
TSC: Trial steering committee
TB: Tuberculosis
ULN: Upper limit of normal
UP: Unanticipated problems
WHO: World Health Organization
Z: Pyrazinamide

## References

[1] Chan AW, Tetzlaff JM, Gøtzsche PC, Altman DG, Mann H, Berlin JA (2013). SPIRIT 2013 explanation and elaboration: guidance for protocols of clinical trials. BMJ.

[2] World Health Organization. Global tuberculosis report 2022. Geneva; 2022. Available from: https://www.who.int/teams/global-tuberculosis-programme/tb-reports/global-tuberculosis-report-2022

[3] Pareek M, Greenaway C, Noori T, Munoz J, Zenner D (2016). The impact of migration on tuberculosis epidemiology and control in high-income countries: a review. BMC Med..

[4] World Health Organization. WHO global lists of high burden countries for tuberculosis (TB), TB/HIV and multidrug/rifampicin-resistant TB (MDR/RR-TB), 2021–2025: background document. Geneva; 2021. Available from: https://apps.who.int/iris/handle/10665/341980

[5] World Health Organization. WHO consolidated guidelines on tuberculosis. Module 4: treatment - drug-resistant tuberculosis treatment, 2022 update [Internet]. World Health Organization. Geneva; 2022. Available from: https://www.who.int/publications/i/item/9789240063129 36630546

[6] World Health Organization. WHO consolidated guidelines on tuberculosis. Module 4: treatment - drug-susceptible tuberculosis treatment, 2022 update. Geneva; 2022. Available from: https://www.who.int/publications/i/item/9789240063129 35727905

[7] Médecins Sans Frontières. DR-TB drugs under the microscope, 5th Edition (ABRIDGED) | Médecins Sans Frontières Access Campaign. 2018. Available from: https://msfaccess.org/dr-tb-drugs-under-microscope-5th-edition

[8] Kroesen VM, Gröschel MI, Martinson N, Zumla A, Maeurer M, van der Werf TS (2017). Non-steroidal anti-inflammatory drugs as host-directed therapy for tuberculosis: a systematic review. Front Immunol.

[9] Zumla A, Rao M, Wallis RS, Kaufmann SHE, Rustomjee R, Mwaba P (2016). Host-directed therapies for infectious diseases: current status, recent progress, and future prospects. Lancet Infect Dis.

[10] Ravimohan S, Kornfeld H, Weissman D, Bisson GP (2018). Tuberculosis and lung damage: from epidemiology to pathophysiology. Eur Respir Rev..

[11] Hortle E, Johnson KE, Johansen MD, Nguyen T, Shavit JA, Britton WJ (2019). Thrombocyte inhibition restores protective immunity to mycobacterial infection in zebrafish. J Infect Dis.

[12] Kroesen VM, Rodríguez-Martínez P, García E, Rosales Y, Díaz J, Martín-Céspedes M (2018). A beneficial effect of low-dose aspirin in a murine model of active tuberculosis. Front Immunol.

[13] Castellucci LA, Cameron C, Le GG, Rodger MA, Coyle D, Wells PS (2013). Efficacy and safety outcomes of oral anticoagulants and antiplatelet drugs in the secondary prevention of venous thromboembolism: systematic review and network meta-analysis. BMJ.

[14] Eisen DP, McBryde ES, Walduck A (2013). Ibuprofen’s sterilizing effects on mycobacterium tuberculosis suggest safe new adjuvant therapies for. J Infect Dis.

[15] World Health Organization. World Health Organization model list of essential medicines – 22nd List, 2021. Geneva; 2021. Available from: https://www.who.int/publications/i/item/WHO-MHP-HPS-EML-2021.02

[16] Aspirin monograph for professionals - Drugs.com. [cited 2023 Feb 7]. Available from: https://www.drugs.com/monograph/aspirin.html

[17] Smith T, Elwood P, Keating C, Rothwell P, Detering E, Freedman A (2014). The Aspirin Foundation Scientific Conference: the history, the present state and the future of aspirin prophylaxis. Ecancermedicalscience..

[18] Rainsford K (2009). Ibuprofen: pharmacology, efficacy and safety. Inflammopharmacology.

[19] Ibuprofen monograph for professionals - Drugs.com. Available from: https://www.drugs.com/monograph/ibuprofen.html

[20] Maitre T, Bonnet M, Calmy A, Raberahona M, Rakotoarivelo RA, Rakotosamimanana N (2022). Intensified tuberculosis treatment to reduce the mortality of HIV-infected and uninfected patients with tuberculosis meningitis (INTENSE-TBM): study protocol for a phase III randomized controlled trial. Trials.

[21] Mai NT, Dobbs N, Phu NH, Colas RA, Thao LT, Thuong NT (2018). A randomised double blind placebo controlled phase 2 trial of adjunctive aspirin for tuberculous meningitis in HIV-uninfected adults. Elife.

[22] Vilaplana C, Marzo E, Tapia G, Diaz J, Garcia V, Cardona PJ (2013). Ibuprofen therapy resulted in significantly decreased tissue bacillary loads and increased survival in a new murine experimental model of active tuberculosis. J Infect Dis.

[23] Davis AG, Wasserman S, Stek C, Maxebengula M, Jason Liang C, Stegmann S (2023). A phase 2a trial of the safety and tolerability of increased dose rifampicin and adjunctive linezolid, with or without aspirin, for human immunodeficiency virus–associated tuberculous meningitis: the LASER-TBM trial. Clin Infect Dis.

[24] Aunsborg JW, Hønge BL, Jespersen S, Rudolf F, Medina C, Correira FG (2020). A clinical score has utility in tuberculosis case-finding among patients with HIV: a feasibility study from Bissau. Int J Infect Dis.

[25] Wejse C, Gustafson P, Nielsen J, Gomes VF, Aaby P, Andersen PL (2008). TBscore: signs and symptoms from tuberculosis patients in a low-resource setting have predictive value and may be used to assess clinical course. Scand J Infect Dis.

[26] Zumla AI, Gillespie SH, Hoelscher M, Philips PPJ, Cole ST, Abubakar I (2014). New antituberculosis drugs, regimens, and adjunct therapies: needs, advances, and future prospects. Lancet Infect Dis.

[27] Nikolayevskyy V, Holicka Y, van Soolingen D, van der Werf MJ, Ködmön C, Surkova E (2021). Impact of the COVID-19 pandemic on tuberculosis laboratory services in Europe. Eur Respir J..

